# Diagnosis and treatment of Rosai-Dorfman disease of the spine: a systematic literature review

**DOI:** 10.1186/s13643-021-01581-0

**Published:** 2021-01-18

**Authors:** Pan-pan Hu, Feng Wei, Xiao-guang Liu, Zhong-jun Liu

**Affiliations:** Department of Orthopaedics and Beijing Key Laboratory of Spinal Disease Research, Peking University Third Hospital, No. 49 North Garden Rd, Haidian District, Beijing, 100191 China

**Keywords:** Spine, Rosai-Dorfman disease, Literature review, Clinical features, Treatment

## Abstract

**Purpose:**

To review and summarize the clinical features, diagnosis, treatment strategies, and prognosis of spinal Rosai-Dorfman disease (RDD).

**Methods:**

RDD is also termed as sinus histiocytosis with massive lymphadenopathy. We searched the databases of PubMed, Elsevier ScienceDirect, SpringerLink, and OVID. The keywords were *Rosai-Dorfman disease* and *spine/central nervous system*. Research articles and case reports with accessibility to full texts regarding spinal RDD were eligible for the inclusion. A total of 62 articles were included, and they contained 69 cases. We extracted the information of interest and analyzed them using SPSS statistics package.

**Results:**

The average age was 33.1 ± 18.3 years. The ratio of males to females was 1.9/1. Overall, 63 cases presented with spine-related symptoms. A total of 27 cases (39.1%) had multi-organ lesions, and 12 cases had records of massive lymphadenopathy. Among 47 cases who first manifested spine-related symptoms, 93.6% were preoperatively misdiagnosed. The disease had a predilection for cervical spine (38.8%) and thoracic spine (40.3%). 62.9% of lesions were dura-based. Surgery remained the mainstream treatment option (78.8%), with or without adjuvant therapies. Total lesion resection was achieved in 34.8% of cases. The rate of lesion recurrence/progression was 19.5%, which was marginally lower for total resection than for non-total resection.

**Conclusion:**

Spinal RDD has no pathognomonic clinical and imaging features. Most cases first present with spine-relevant symptoms. Massive lymphadenopathy is not common, but a tendency for multi-organ involvement should be considered. Spinal RDD has a high recurrence rate; thus, total resection is the treatment of choice. Adjuvant therapies are indicated for multi-organ lesions and residual lesions. A wait and watch strategy is recommended for asymptomatic patients. Herein, a workflow of diagnosis and treatment of the spinal RDD is established.

**Supplementary Information:**

The online version contains supplementary material available at 10.1186/s13643-021-01581-0.

## Background

Rosai-Dorfman disease (RDD) is a benign disorder, and it is also termed sinus histiocytosis with massive lymphadenopathy [1–3]. Previous etiological studies did not arrive at any firm conclusions. Viral infections, autoimmune conditions, or gene mutations were reported to be related to the development of RDD [3–8]. There were also reports of family cases [9]. Symptomatically, painless neck, axillary, and/or inguinal lymph node enlargement is present in about 80% of cases. A few patients have fever, weight loss, anemia, and malaise [1, 3]. Over 40% of cases have extranodal lesions, and the most commonly involved organs are the skin, respiratory tract, orbit, paranasal sinuses, heart, liver and kidney, bone, and central nervous system [1–3, 9, 10]. The disease has a tendency for recurrence and involvement of multiple organs; Mayo clinic has referred to this condition as a histiocytic neoplasm [3].

It is estimated that 0.6–1% of RDD cases have isolated or concurrent spinal lesions [2, 3, 9–12]. The bone, dura, and cord parenchyma are all nidus candidates. Spinal RDD has no pathognomonic clinical and imaging features. The disorder is often misdiagnosed as infection, spinal tuberculosis, extradural hematoma, meningioma, and other primary or metastatic tumors. It is hardly possible to make the diagnosis unless histopathological and immunohistochemical examinations are performed. There are no consensus guidelines for the treatment of spinal RDD. Treatment regimens are usually prescribed based on personal experience rather than high-level medical evidence. Therefore, we performed a systematic literature review specifically on spinal RDD. This study provides the following referential contents to peers: (a) clinical features of the disease; (b) imaging traits of RDD lesions; (c) summary of current treatment strategies and outcomes; (d) prognosis and possible relevant clinical and therapeutic factors.

## Materials and methods

### Literature search

A literature search was performed using the following databases: PubMed, Elsevier ScienceDirect, SpringerLink, and OVID. The search strategy for PubMed was presented as follows: Mesh term #1: (Rosai-Dorfman)OR (sinus histiocytosis with massive lymphadenopathy): ti, ab, kw; Mesh term #2: (spine) OR (central nervous system). #1 AND #2. We set no limitation regarding the searching fields and date. The language was set as English. All studies retrieved were saved in the software package of EndNote X7. All retrieved literatures were carefully screened. The inclusion criteria were (1) literature regarding the cases with spinal RDD; (2) full texts were available; (3) publication types of research articles and case reports; (4) literature with complete records of patients’ demographic information, clinical, and imaging manifestations; (5) references of the eligible articles were also screened. The exclusion criteria were (1) articles in non-English languages; (2) literature with no specific cases recorded; (3) repeated literatures or same cases by same authors in different literatures. The searching, literatures screening, and selecting were performed by PH and FW independently. The data extraction was also performed independently by the two authors. The results were checked by the other authors. All eligible articles met the inclusion criteria, and they did not fulfill the exclusion criteria (Table 1).

**Table 1:** The inclusion and exclusion criteria of the study

Inclusion criteria	
a. Cases with spinal lesions, whether symptomatic or not	
b. Full texts were availed	
c. Publication types of research articles and case reports	
d.Literature with complete records of patients’ demographic information, clinical and imaging manifestations	
e. References of the eligible articles were also screened	
Exclusion criteria	
a. Articles in non-English languages	
b. Reviews, comments, or other articles wherein no cases were recorded	
c. Duplicated literature or same cases in different articles	

### Risk of bias

Considering the included studies were case reports, which were ranked as level 4 evidence, an 8-item JBI Critical Appraisal Checklist for Case Reports was employed to assess the risk of bias (ROB). If the answers for all the 8 items were YES, this study was appraised as low ROB; otherwise, the ROB was high. The appraisal of the included articles was undertaken independently by PH and WF. When disagreement occurred between the two appraisal results, they were then discussed and arbitrated by the all authors.

### Data collection

The full text of the included articles was studied. The following information was extracted: (1) clinical features: genders, age, symptomatic presentations (asymptomatic, neurological deficits and/or local pain); (2) imaging features: vertebral segments (cervical, thoracic, lumbar, and/or sacrum), sites of the niduses (intrabony, dura-based, and/orintramedullary); (3) systemic involvement: lesions of lymph nodes or other organs; (4) laboratory test results; (5) treatment regimens: wait and watch, surgery, and/or non-surgery therapies; (6) prognosis and therapeutic outcomes. All the data were then transcribed onto an Excel sheet (see Supplementary Table 1). For data not provided in original articles, we left the slots vacant.

### Statistical analysis

Data analyses were performed using IBM SPSS statistics for Windows Version 20 (IBM Corp., Armonk, NY, USA). Lilliefors test, which is an adaptation of the Kolmogorov-Smirnov test, was used to examine whether the data were normally distributed. Data presentation was provided in the forms of percentages, mean ± standard deviation, or median (quartile range). Two-tailed unpaired Student’s *t* test and two-tailed Pearson’s *χ*2 test (or Fisher’s exact test) were utilized to make a comparison between different groups. Statistical significance was set at 0.05.

## Results

**Fig. 1:**
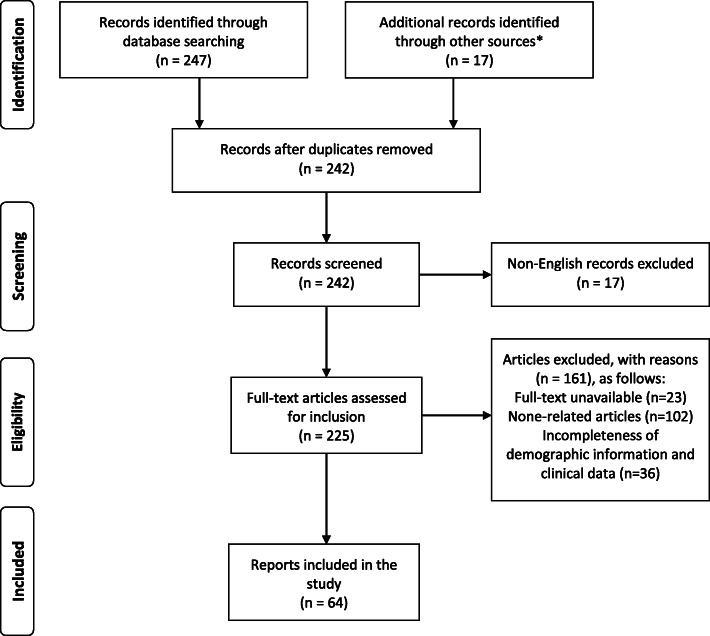
Flow diagram of the literature search, screening, and inclusion in the present study. *The other source besides the searched databases was references of the included reports

A total of 62 articles were included in the study, and they contained 69 cases of spinal RDD (Fig. 1 and Supplementary Table 1) [11–72]. These articles were published from 1976 to 2020, and 42 articles (67.7%, 42/62) were published after 2010. Twelve included studies had high ROB [13, 16–18, 31, 34, 46, 48, 49, 53, 56, 57], whilst 50 other studies had low ROB. The cohort included 45 males and 24 females, at a ratio of 1.9/1. The average age was 33.1 ± 18.3 years. Follow-up information was documented in 52 cases, and the median period was 12.0 (11.8) months. The median course before consultation and admission was 2.1 (5.0) months (Table 2).

**Table 2:** Presentation of basic data of the included spinal Rosai-Dorfman cases^a^

Item	Value
Age, mean ± standard deviation	(33.1 ± 18.3) years
Course, M(QR)	2.1(5.0) months
Follow-up period, M(QR)	12.0(11.8) months
Spine-related symptoms	63(91.3%)
Focal pain at the spinal lesions	31(44.9%)
Myelopathy/radiculopathy	54(78.3%)
Lymph nodes enlargement	12(17.4%)
Multiple organ involvement	27(39.1%)
Abnormal laboratory tests	17(24.6%)

M(QR) stands for median(quartile range)

^a^Only data that were originally documented were included in this statistical analysis

A total of 63 cases (91.3%, 63/69) manifested spine-related symptoms. A total of 54 cases (78.3%, 54/69) suffered from myelopathy or radiculopathy, and 31 cases (44.9%, 31/69) complained of focal pain in the lesions. Multiple organ involvement was reported in 27 cases (39.1%, 27/69), and massive lymph nodes were found in 12 cases (17.4%, 12/69). Abnormal results of laboratory tests, such as elevated white blood cell count, C-reactive protein, and erythrocyte sedimentation rate, were described in 17 cases (24.6%, 17/69) (Table 2).

The initial symptoms of 47 cases (68.1%, 47/69) were spine-related; among them, 10 cases (21.3%, 10/47) were screened for coexisting niduses at other organs by imaging, and 6 cases (12.8%, 6/47) had lymph node enlargement. Only 3 cases (6.4%, 3/47) were diagnosed correctly, while the other 93.6% of cases (44/47) were misdiagnosed before postoperative pathological examinations. A total of 22 cases first presented with painless lymph node enlargement or other organ-related symptoms; among them, 16 cases (72.7%, 16/22) developed spinal lesions and symptoms afterward.

**Fig. 2:**
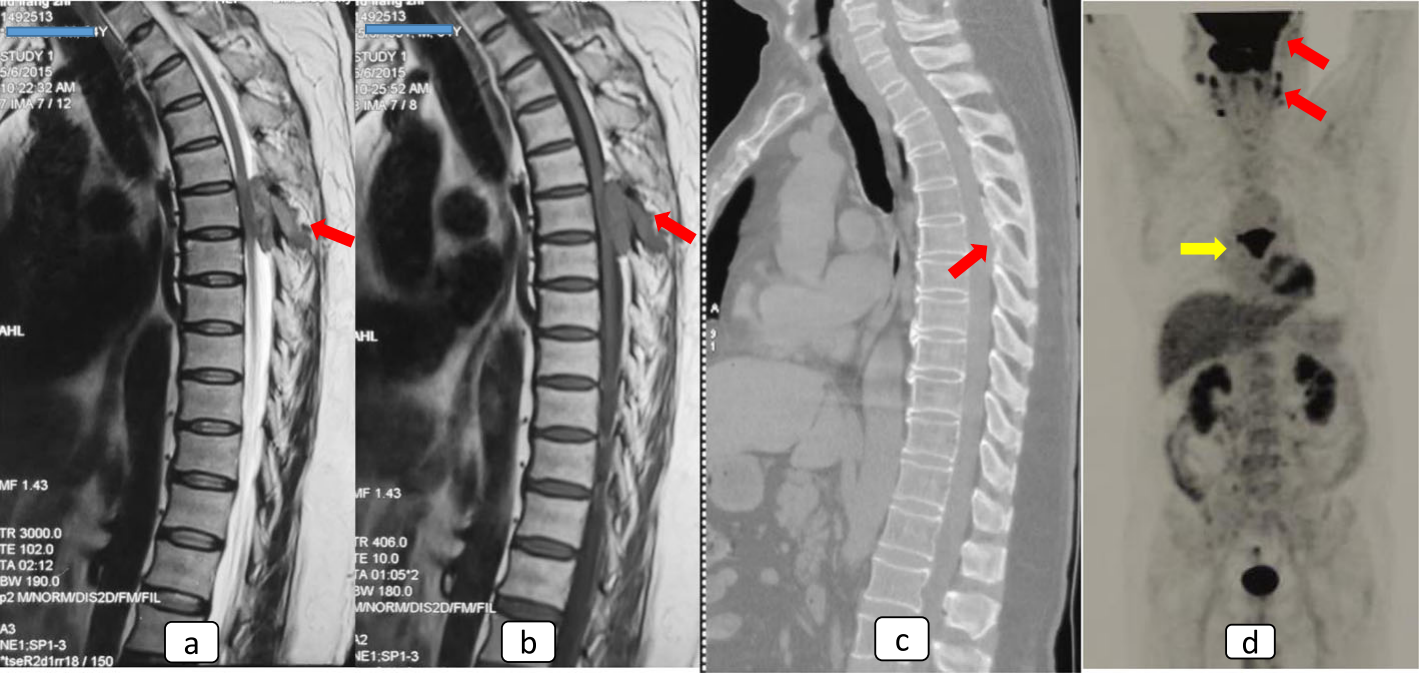
The imaging presentation of a case with spinal RDD. MRI scans manifested a T1 isointense (**a**) and T2 slightly hyperintense (**b**) extradural lesion on Th6. The spinous process was also involved (arrows on **a** and **b**). CT scan revealed an osteolytic lesion, but the cortex seemed intact (arrow on **c**). PET/CT scan was arranged. Paranasal lesions and cervical lymph node lesions were also found and the lesions manifested a high uptake of 18-FDG (red arrows on **d**)

Imaging work-ups revealed that the lesion manifested T1-weighted isointensity to the cord and T2-weighted slight hyperintensity on MRI scans (Fig. 2a, b). The bony lesions were osteolytic and non-expansile and were located inside cancellous bones (Fig. 2c). PET/CT could reveal multiple organ lesions, which were moderate to high uptake of 18-fluorodeoxyglucose (Fig. 2d).

The lesion sites of spinal RDD were documented in 67 cases; among them, 26 cases (38.8%, 26/67) developed RDD lesions in the cervical spine and 27 cases (40.3%, 27/67) developed RDD lesions in the thoracic spine (Table 3). Seven cases (10.4%, 7/67) had lesions in multiple spinal segments. The nidus sites and lesion bases were described in 62 cases. Intracanal dura-based lesions were found in 39 cases (62.9%, 39/62), whereas the rates of intramedullary and intrabony lesions were 9.7% (6/62) and 12.9% (8/62), respectively. Altogether, there were 53 cases (85.5%, 53/62) with lesions focally constrained to one spinal component, namely bone, dura, or cord parenchyma. A total of 9 cases (14.5%, 9/62) had focally concurrent lesions invading multiple spinal components, for example, bone and dura (Table 3).

**Table 3:** Summary of lesion sites on spinal segments and lesion bases

Spinal segments (*n* = 67)			Lesion bases/origins (*n* = 62)		
	*Count*	*Percentage*		*Count*	*Percentage*
Cervical	26	38.80%	Intramedullary	6	9.70%
Thoracic	27	40.30%	Intrabony	8	12.90%
Lumbar	4	6.00%	Dura-based	39	62.90%
Sacrum	3	4.50%	Concurrent	9	14.50%
Multi-segment lesions	7	10.40%			

The diagnoses were made based on histopathological and immunochemical examinations in all the included cases, rather than on clinical and imaging manifestations. The histopathological features were histiocytic cells in a fibrous background, with an infiltration of lymphocytes and plasma cells, and emperipolesis of surrounding cells into histiocytic cells. The immunohistochemical phenotype was CD68 and S100 positive and CD1a negative; this phenotype is specific and diagnostic, according to the included reports.

**Fig. 3:**
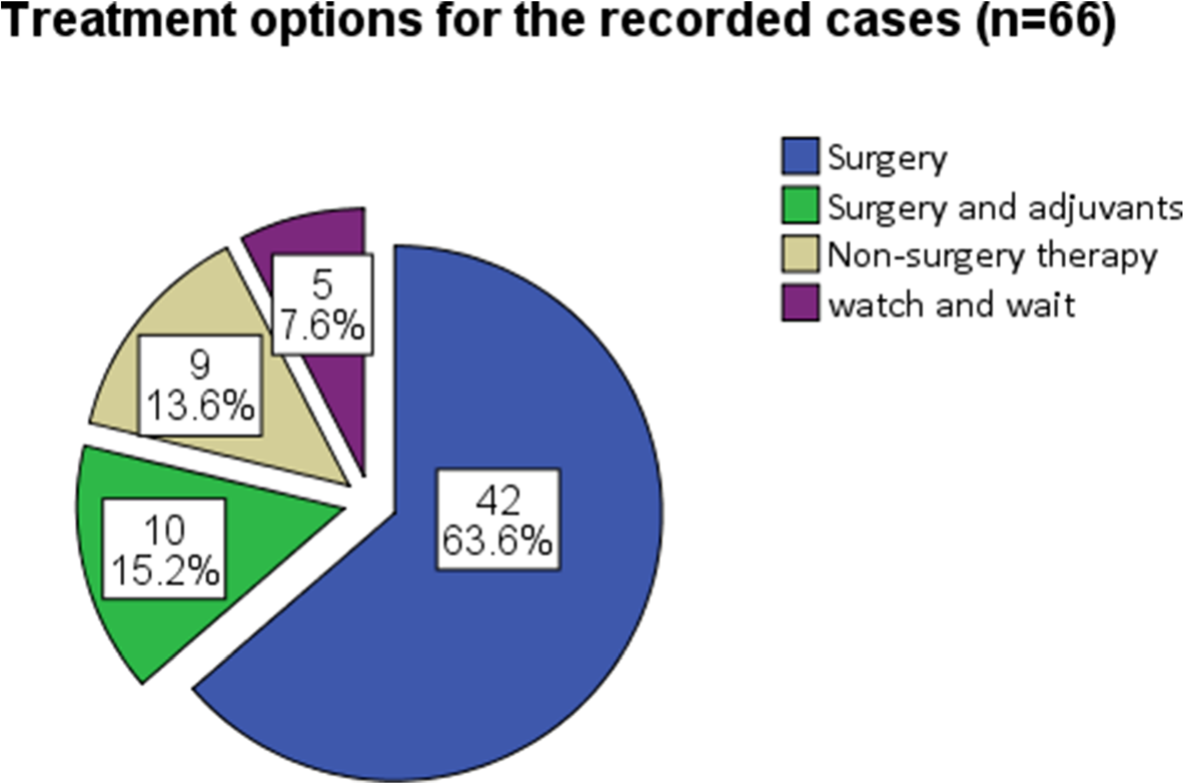
Treatment options for 66 cases, whose treatment regimens were recorded in the original literature. Non-surgery therapies include radiotherapy, steroids and chemotherapy

Treatment regimens were reported in 66 cases. A total of 42 cases (63.6%, 42/66) underwent surgeries, 10 cases (15.2%, 10/66) received surgeries and adjuvant therapies, and 9 cases (13.6%, 9/66) received radiotherapy, steroids, and/or chemotherapy without surgeries (Fig. 3). A total of 5 cases (7.6%, 5/66) received no specific treatment but a wait and watch strategy [17, 46, 48, 64, 69]. Among the 52 cases receiving surgeries, total lesion resection was achieved in 23 cases (44.2%), and 29 cases (55.8%) underwent decompression surgeries with or without partial lesion resection.

A total of 54 cases reported follow-up data (Table 4). A total of 44 cases (81.5%, 44/54) reported no recurrence or stable residual lesions, whereas 10 cases (19.5%, 10/54) experienced recurrence or progression of spinal lesions. Lesions constrained to the dura, intrabony region, or intramedullary region were correlated with lower recurrence and progression rates than focally unconstrained lesions (*p* = 0.033). Patients with systemic multiple lesions had marginally higher recurrence and progression rates than patients with isolated spinal lesions (*p* = 0.346). Among those who underwent surgery, the rate of recurrence/progression was 17.8% and 22.2% for non-surgery cases (*p* = 0.754). The recurrence and progression rates for cases after total resection and partial resection were 9.1% and 26.1%, respectively, with no significant difference (*p* = 0.243). For those who received radiotherapy, steroids, and/or chemotherapy, the recurrence/progression rate was 28.6% and 15.0% for the patients who did not receive the above therapies, with no significant difference (*p* = 0.261).

**Table 4:** Correlation analysis of lesion recurrence or progression with clinical and therapeutic factors

Clinical and therapeutic factors^*^	Recurrence and/or progression of spinal lesions		
	Yes	No	*p* value
Age (*n* = 54)	36.2 ± 21.8	34.1 ± 18.5	0.758
Focal lesion conditions (*n* = 51)			
Focally constrained	5(11.4%)	39(88.6%)	0.033^#^
Focally concurrent	3(42.9%)	4(57.1%)	
Involved organs (*n* = 33)			
Single	1(6.7%)	14(93.3%)	0.346
Multiple	4(22.2%)	14(77.8%)	
Treatment options (*n* = 54)			
Surgery	8(17.8%)	37(82.2%)	0.754
Non-surgery	2(22.2%)	7(77.8%)	
Surgical strategies (*n* = 45)			
Total resection	2(9.1%)	20(90.9%)	0.243
Non-total resection	6(26.1%)	17(73.9%)	
Non-surgery therapies (*n* = 54)			
Yes	4(28.6%)	10(71.4%)	0.261
No	6(15.0%)	34(85.0%)	

Non-surgery therapies include radiotherapy, steroids and/or chemotherapy. Focally constrained lesions mean isolated lesions focally constrained to the dura, intrabony region, or intramedullary region. Focally concurrent lesions mean lesions invading multiple spinal components

*Only data documented in the original literature were included

^#^Statistically significant at *p* < 0.05, two-tailed Pearson’s chi-squared test

**Fig. 4:**
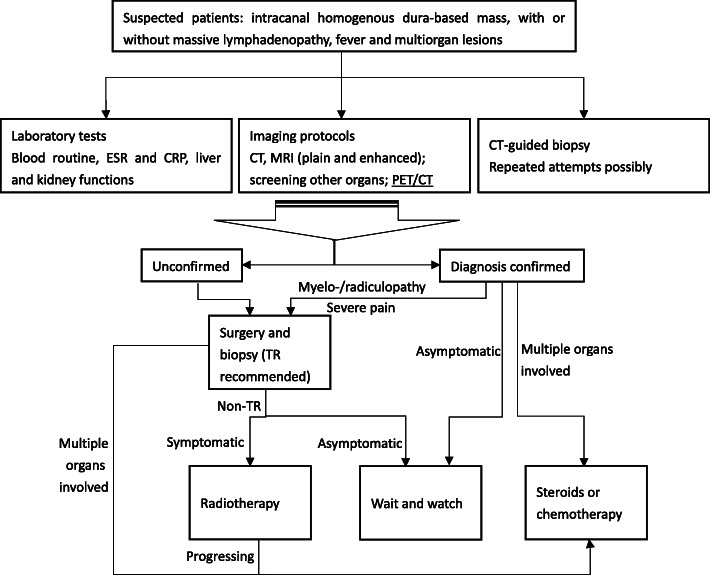
A preliminarily established diagnosis and treatment algorithm for spinal Rosai-Dorfman disease. ESR stands for erythrocyte sedimentation rate; CRP, C-reactive protein; CT, computed tomography; MRI, magnetic resonance imaging; PET/CT, positron emission tomography/computed tomography; TR, total resection

Based on the review and analysis of the data regarding on the clinical symptoms, imaging work-ups, and treatment and their outcomes, a preliminary algorithm was proposed for the diagnosis and treatment of the spinal RDD (Fig. 4).

## Discussion

Spinal RDD poses a great challenge for the diagnosis and treatment due to a lack of mature guidelines in clinical practice. According to our study, 93.6% of cases whose initial symptoms were spine-related could not be correctly recognized until postoperative pathological examination. Computed tomography (CT)-guided biopsy results were recorded in six cases; three cases (50.0%) failed to make a definite and correct diagnosis [17, 24, 31, 34, 57, 62]. The failure rate was relatively high in the included reports. The possible reasons were the failure to harvest the target specimen and failure to recognize it on cellular smears. The features of cellular smear are not diagnostic and cannot be differentiated from Langerhans cell histiocytosis, reactive lymphadenitis, hemophagocytic syndrome, and malignant lymphoma [24, 57].

Spinal RDD has a predilection for young and middle-aged people, with an average age of 33.1 years and 47.8% (33/69) cases aged between 18 and 45 years. The study noted gender variation with a predominance of males (1.9/1). Spinal RDD has no specific and pathognomonic symptoms, although 91.3% of the included cases manifested spine-relevant symptoms. Previous literature reported that about 80% RDD patients have painless massive neck or axillary lymphadenopathy [1, 3, 19, 21]. However, this study found the records of lymph node enlargement in 17.4% of cases, which was significantly lower than that in the previous literature (Table 2). It implies that spinal RDD might have some unique pathological features.

We found that the lesions mostly occurred in the cervical and thoracic spine (79.1%) and that 62.9% of lesions were dura based. The imaging features of RDD are not specific and diagnostic. It is difficult to differentiate this condition from spinal infections, meningioma, lymphoma and other primary or metastatic tumors, chronic inflammation, extradural hematoma, and other histiocytoses, by merely relying on clinical and imaging manifestations.

RDD has a tendency to invade multiple organs [1–3]. We noted reports of deaths due to lesions in other organs [43]. In addition, this study found that the recurrence rate of isolated spinal lesions was marginally lower than that of multi-organ lesions (6.7% versus 22.2%). Therefore, it is necessary to screen all possible lesions in other organs when dealing with spinal RDD patients. Mayo clinic reported a consensus on the diagnosis of systemic RDD [3]. It recommended positron emission tomography/computed tomography (PET/CT) to rule out systemic multiple lesions in each patient. PET/CT is helpful to screen whole-body lymph nodes and discover some uncommon and small lesions, for example, paranasal sinuses (Fig. 2d). In the included case series, only four cases had the records of PET/CT. This might explain the significantly lower rate of lymph node enlargement in included case series in this study (Table 2). We also noticed that many included cases presented with rapidly exacerbating myelopathy, and they were asked to undergo urgent cord decompression surgeries, leaving no time for PET/CT. In this setting, we recommend imaging and ultrasonic examinations of vital organs in the intracranial and abdominal cavity.

This study explored the relationship between focal conditions of the lesions and prognosis. We found that focally unconstrained lesions were correlated with a higher recurrence rate (Table 4). Generally, it is difficult to completely resect unconstrained lesions, and residual lesions have a potential for progression and recurrence. More importantly, focal involvement of multiple spinal components implied that these lesions were able to infiltrate through the anatomical septa and possessed stronger aggressiveness. This finding may provide us an important reference when dealing with spinal RDD.

RDD is benign histiocytosis, and some lesions can resolve spontaneously [47, 65]. Pulsoni et al. reviewed 40 cases of untreated RDD and found 82% remitted spontaneously [73]. Rittner and colleagues reported a case with multiple whole-body osseous lesions, including the spine, and it was treated with non-steroidal anti-inflammation drugs [47]. After 6 months, most of the lesions resolved spontaneously. Therefore, wait and watch is recommended for patients without symptoms and signs of spinal instability and neurological impairment.

However, for symptomatic patients, for whom cord decompression and restoration of spinal stability are indicated, or malignancy cannot be ruled out, surgery is the treatment of choice whenever feasible. Theoretically, total resection is a better choice, considering the tendency of the lesions to recur (Table 4) [3, 14, 15, 29, 30, 32, 43, 44, 49, 51]. This study found that the recurrence rate of total resection was marginally lower than that of non-total resection (9.1% versus 26.1%). Given a longer follow-up period (the median value of the included cases was 12 months, see Supplementary Table 1), we infer that the difference of the recurrence rates between the total and non-total resection groups would be larger and more statistically significant. Therefore, total resection is the recommended option, especially for those isolated, primary spinal RDD lesions. When the lesions are not completely resectable or systemic lesions are present, adjuvant therapies are indicated. Adjuvant therapies mainly include radiotherapy, steroids, and chemotherapy. However, they were not proven beneficial in this study (Table 4). Radiotherapy has provided excellent locoregional control for isolated lesions, but incompetent for extensive systemic involvement. Chemotherapy regimens were usually prescribed based on personal experience rather than high-level evidence, and its efficacy was not verified in the most patients [73]. The efficacy of steroids was not supported in the case series of this study, either, especially for aged ones or patients with multiple organs involvement [11, 14, 31, 44, 45, 62]. Thus, surgical resection, with or without adjuvant therapies, remains as a primary modality for spinal RDD when therapeutic interventions are indicated.

Based on a comprehensive review of the literature on spinal RDD, we established a preliminary workflow for the diagnosis and treatment of this disease, which may provide some assistance to our peers. For patients suspicious of RDD, laboratory tests of inflammatory indexes and imaging work-ups including CT and MRI are imperative. PET/CT is also strongly recommended, which can rule out systemic lesions and, especially, the involvement of superior lymph nodes. Considering the fact that imaging modalities are not diagnostic, CT-guided biopsy is often necessary. Surgery is the treatment of choice for the patients who present with symptoms of neurological deficits and refractory local pain. Adjuvant therapies, including steroids, chemotherapy, and radiotherapy, are indicated for progressive lesions and patients with systemic involvement. For the patients without neurological deficits and signs of spinal instability, a wait and watch strategy shall be adopted. However, this study found that the rate of lesion recurrence/progression in the spine was as high as 19.5%, implying that the current treatment strategies for spinal RDD still need to be improved. Thus, high-level clinical research studies are required to eventually build a standard and more effective treatment guideline.

## Limitations

Firstly, the included literatures in this study were mostly case reports, which were referred to low-quality evidences. This can influence the quality of this study. Secondly, there may be risk of inclusion bias due to searching strategies and the availability to some literature. Thirdly, some of included literature did not have the records of all the data of interest, which might influence statistical analysis and the interpretation of the results.

## Conclusions

The diagnosis and treatment of spinal RDD remain a huge challenge. It has no pathognomonic clinical and imaging features. Most cases initially present with spine-related symptoms. Only a small number of cases have massive lymphadenopathy, fever, and other constitutional symptoms. The lesions have a predilection for the cervical and thoracic spine, and they are mostly dura based. PET/CT is helpful to discover whole-body lesions and can provide some evidence for the diagnosis. Generally, pathological examinations are an exclusive way to make the diagnosis. The rate of recurrence and new occurrence in other vertebrae was as high as 20%; thus, total lesion resection is perceived as the ideal surgical option. Radiotherapy, steroids, and/or chemotherapy are utilized individually or in cases with residual lesions. Some cases can remit spontaneously, so a wait and watch strategy is recommended in patients without symptoms and signs of spinal instability and neurological impairment.

## Supplementary Information

**Additional file 1: Supplementary Table 1.** Detailed information of the included cases with spinal Rosai-Dorfman disease in this study.

## Data Availability

We provide all supporting data in Supplementary Table 1.

## Abbreviations

RDD: Rosai-Dorfman disease
CT: Computed tomography
PET/CT: Positron emission tomography/computed tomography
FDG: Fluorodeoxyglucose
ESR: Erythrocyte sedimentation rate
CRP: C-reactive protein
MRI: Magnetic resonance imaging
TR: Total resection
