# Supplementary material for: Diagnosis and treatment of Rosai-Dorfman disease of the spine: a systematic literature review
Source: Syst Rev. 2021 Jan 18;10:31. doi: 10.1186/s13643-021-01581-0 (PMC7814441; doi:10.1186/s13643-021-01581-0)
Supplement: Supplementary file 1 — Additional file 1: Supplementary Table 1. Detailed information of the included cases with spinal Rosai-Dorfman disease in this study. [file 13643_2021_1581_MOESM1_ESM.docx]

**Supplementary table 1** Detailed information of the included cases with spinal Rosai-Dorfman disease in this study

| Literature | Age/gender | spine-related symptoms | other organs involvement | lymphadenopathy | Laboratory tests | Imaging finding | Treatment | Follow-up and Recurrence |
| --- | --- | --- | --- | --- | --- | --- | --- | --- |
| Baassiri W, *et al* | 54/male | Neck pain and myelopathy for 6 mon | *Not mentioned* | *Not mentioned* | *Not mentioned* | Craniocervical junction, intradural lesion | Near-total resection, radiotherapy | *Not mentioned* |
| Qin G, *et al* | 43/male | Myelopathy for 5 mon | *Not mentioned* | *Not mentioned* | *Not mentioned* | Thoracic, intradural lesion | Laminectomy and biopsy | 2 year later, new intracranial lesions |
| Lin C, *et al* | 32/male | Low back pain and myelopathy for 3 mon | Multiple organs involvement | *Not mentioned* | Leukocytosis | Thoracic, dural and vertebral lesion | Laminectomy and biopsy | 3 years later, recurrent |
| Kumaran SP, *et al* | 45/male | Myelopathy for 3 mon | *Not mentioned* | *Not mentioned* | *Not mentioned* | *Not mentioned* | *Not mentioned* | *Not mentioned* |
| Kong Z, *et al* | 10/male | No spine-related symptoms | Multiple organs involvement | No | *Not mentioned* | Thoracic, processus transversus | *Not mentioned* | *Not mentioned* |
| Joshi SS, *et al* | 36/male | Low back pain and radiculopathy | Multiple organs involvement | No | *Not mentioned* | Sacrum and thoracic, bony lesion | *Not mentioned* | *Not mentioned* |
| Li Y, *et al* | 28/male | Back pain and myelopathy for 1 year | *Not mentioned* | No | Normal | Thoracic, extradural and vertebra | Laminectomy and lesion resection | 6 months, no recurrence |
| Chhabria BA, *et al* | 19/female | Low back pain and conus-cauda syndrome for 3 months | No | No | Elevated ESR and anemia | Lumbar, dura-based | Subtotal resection | Stable |
| Xu H, *et al* | 34/male | Myelopathy for 1 month | *Not mentioned* | No | Elevated ESR | Thoracic, epidural | Laminectomy and total resection | 5m, no recurrence |
| Karthigeyan M, *et al* | 21/male | Myelopathy for 3 months | No | No | *Not mentioned* | Craniocervical junction, dura-baed | Total resection | 6 months, no recurrence |
| Carrasco-García de León S, *et al* | 28/male | Myelopathy for 1 week | Multiple involvement | *Not mentioned* | *Not mentioned* | Craniocervical junction, intracanal | Total resection, steroids | 4 years, no recurrence |
| Tu J, *et al* | 41/male | Back for 1 year | *Not mentioned* | No | *Not mentioned* | Thoracic, vertebrae, intracanal and paravertebral | Total resection | 3 months, no recurrence |
| Tripathi R, *et al* | 7.5/female | Radiculopathy | Multiple organs involvement | Yes | *Not mentioned* | Lumbar, dura-based | Steroids and immunoglobulin | 7 months, stable |
| Rocha-Maguey J, *et al* | 27/female | Myelopathy for 2 months | No | No | Elevated ESR | Cervical, intramedullary | Total resection | 6 months, no recurrence |
| Igrutinovic Z, *et al* | 14/female | Cervical pain for 1 month | No | No | Normal | Cervical, vertebral fracture | Corpectomy | 2 years, stable |
| Huang BY, *et al* | 55/male | Myelopathy for 1 month | No | No | Normal | Thoracic, epidural | Subtotal resection | 6 months, no recurrence |
| Huang BY, *et al* | 40/male | Neck pain and myelopathy for 1 year | No | No | Leukocytosis | Cervical, epidural | Liminectomy and total resection | 18 months, no recurrence |
| Huang BY, *et al* | 14/female | Radiculopathy for 4 months | No | No | Normal | Sacrum, vertebra | Laminectomy and total resection | 12 months, no recurrence |
| Huang BY, *et al* | 43/male | Neck pain and myelopathy for 8 months | No | No | Normal | Cervical, epidural | Laminectomy and total resection | 12 months, no recurrence |
| Huang BY, *et al* | 12/female | Myelopathy for 1 month | No | No | Normal | Cervical, intramedullary | Total resection | 12 months, no recurrence |
| de Oliveira Lima GL, *et al* | 50/female | Myelopathy for 20 days | *Not mentioned* | No | *Not mentioned* | Thoracic and lumbar, intradural lesion | Laminectomy and resection | Progression |
| Sciacca S, *et al* | 75/female | Myelopathy | No | No | *Not mentioned* | Thoracic, epidural and vertebra | Hemi-laminectomy and partial resection, steroids | *Not mentioned* |
| Fu X, *et al* | 25/female | Neck pain and myelopathy for 6 months | No | No | Normal | Cervical, intradural | Laminectomy and total resection | 12 months, no recurrence |
| Tian Y, *et al* | 40/male | Neck pain and myelopathy for 6months | *Not mentioned* | Yes | *Not mentioned* | Cervical, epidural | Total resection | 9 months, died from other diseases |
| Tian Y, *et al* | 43/male | Shoulder pain and myelopathy for 8 months | *Not mentioned* | No | *Not mentioned* | Cervical, intracanal | Total resection | 22 months, recurrence |
| Mannelli L, *et al* | 49/female | No spine-related symptoms | Multiple organs involvement | *Not mentioned* | *Not mentioned* | Cervical, epidural and vertebra | *Not mentioned* | *Not mentioned* |
| Kozak B, *et al* | 26/male | Myelopathy for 2 weeks | No | *Not mentioned* | Elevated ESR | Thoracic, epidural | Laminectomy and resection | 18 months, no recurrence |
| Sandoval-Sus J, *et al* | 32/female | Neck stiffness | Multiple organs involvement | No | *Not mentioned* | Craniocerebral junction, intracanal | Radiotherapy and chemotherapy | 3 years, stable |
| Sandoval-Sus J, *et al* | 53/male | Myelopathy for 3 months | Multiple organs involvement | No | *Not mentioned* | Cervical and thoracic, intramedullary | Steroids and radiotherapy | Progression |
| Wu L, *et al* | 43/male | Back pain and myelopathy for 8 months | *Not mentioned* | No | *Not mentioned* | Cervical, intradural | Laminotomy and total resection | 18 months, no recurrence |
| Kim DY, *et al* | 15/male | Back pain for 6 months | No | No | Normal | Thoracic, vertebral fracture | Non-total resection | 1 year, stable |
| El Molla M, *et al* | 76/male | Myelopathy for 10 weeks | *Not mentioned* | No | *Not mentioned* | Cervical, intramedullary | Laminectomy and total resection | 12 months, no recurrence |
| Parmar V, *et al* | 64/male | Neck pain and radiculopathy for 5 weeks | Multiple organs involvement | No | Normal | Cervical, intradural | Laminectomy and resection | 9 months, no recurrence |
| Zhu F, *et al* | 53/male | Myelopathy for 1 year | *Not mentioned* | Yes | Leukocytosis and neutrophilia | Thoracic, intrabony and intracanal | Surgery | 1 year, stable |
| Yao K, *et al* | 12/female | Myelopathy for 2 weeks | *Not mentioned* | *Not mentioned* | Normal | Cervical, intramedullary | Total resection | 1.5 years, no recurrence |
| Roy C, *et al* | 32/male | Low back pain and myelopathy for 2 months | No | No | *Not mentioned* | Thoracolumbar junction, extradural and intrabony | Laminectomy and total resection | 4 months, no recurrence |
| Chen CW, *et al* | 16/female | Myelopathy | *Not mentioned* | No | Leukocytosis | Thoracic, intradural | Costotransversectomy and total resection | 2 years, no recurrence |
| Antuna Ramos A, *et al* | 10/female | Back pain and myelopathy for 1 month | Multiple organs involvement | *Not mentioned* | *Not mentioned* | Thoracic, intradural | Two-staged total resection, steroids and radiotherapy | 5 months, died from intracranial lesions |
| Warrier R, *et al* | 6/male | No spine-related symptoms | Multiple organs involvement | Yes | *Not mentioned* | Spine | Radiotherapy, methylprednisone and chemotherapy | Progression |
| Zhu H, *et al* | 58/male | Back pain | *Not mentioned* | *Not mentioned* | *Not mentioned* | Thoracic, dura-based | Rachitomy | *Not mentioned* |
| Rittner RE, *et al* | 15/male | Fever, back pain and iliac crest pain, weight loss | Multiple whole-body bony lesions | No | Elevated ESR and CRP, anemia and leukocytosis | Thoracic, vertebra | Pain killers | 6 months, lesions remission |
| Raslan OA, *et al* | 54/male | No spine-related symptoms | Multiple organs involvement | *Not mentioned* | *Not mentioned* | Craniocervical junction, intracanal | Steroids | *Not mentioned* |
| Raslan OA, *et al* | 57/female | Back and chest pain | Multiple organs involvement | *Not mentioned* | *Not mentioned* | Thoracic, vertebral and epidural | Radiotherapy | *Not mentioned* |
| Ambekar S, *et al* | 37/female | Myelopathy | Multiple organs involvement | *Not mentioned* | Elevated ESR | Cervical, dura-based | Lesion resection | *Not mentioned* |
| Maiti TK, *et al* | 19/female | Neck pain and myelopathy for 2 months | *Not mentioned* | *Not mentioned* | Leukocytosis, anemia and elevated ESR | Cervical, extradural | Laminectomy and lesion resection | 1 year, recurrence |
| Wang Y, *et al* | 58/male | Myelopathy for 3 months | No | *Not mentioned* | *Not mentioned* | Thoracic, intradural | Laminectomy and lesion resection | 7 months, no recurrence |
| Abou-Zeid AH, *et al* | 24/male | Back pain and myelopathy for 1 week | *Not mentioned* | No | Leukocytosis | Thoracic, epidural | Laminectomy and biopsy | 6 months, new lesion on other vertebra level |
| Deng X, *et al* | 38/male | No spine-related symptoms | Multiple organs involvement | *Not mentioned* | Normal | Cervical, intracanal | *Not mentioned* | *Not mentioned* |
| Konca C, *et al* | 36/male | Back pain and myelopathy for 2 months | Multiple organs involvement | Yes | Anemia, leukocytosis and elevated ESR | Thoracic and lumbar, epidural | Steroids and chemotherapy | 9 months, no recurrence |
| Raslan OA, *et al* | 50/male | Radiculopathy | Multiple organs involvement | *Not mentioned* | *Not mentioned* | Lumbar, subdural | Surgery | 8 months, new lesion on other segment |
| Gupta P, *et al* | 16/male | Back pain and myelopathy for 3 months | No | No | *Not mentioned* | Thoracic, vertebral and epidural | *Not mentioned* | *Not mentioned* |
| Jing X, *et al* | 51/female | Back and myelopathy for 1 month | No | Yes | *Not mentioned* | Thoracic | Semihemilaminectomy and transpedicular vertebral body decompression | *Not mentioned* |
| Dran G, *et al* | 17/male | Myelopathy for 1 month | *Not mentioned* | Yes | Normal | Thoracic and cervical, dura-based | Laminotomy and total resection | 18 months, no recurrence |
| Huang YC, *et al* | 31/female | Back pain and myelopathy for 3 months | Multiple organs involvement | No | *Not mentioned* | Thoracic, epidural | Laminectomy and total resection | 24 months, no recurrence |
| Seyednejad F, *et al* | 43/female | Myelopathy for 1 month | Multiple organs involvement | *Not mentioned* | *Not mentioned* | Cervical, intradural | Radiotherapy and steroids | 40 months, no recurrence |
| Hargett C, *et al* | 29/female | Back pain and myelopathy for 1 month | *Not mentioned* | *Not mentioned* | *Not mentioned* | Thoracic, epidural | Total resection, steroids and radiotherapy | 48 months, new lymphadenopathy |
| Tubbs RS, *et al* | 13/male | Neck pain for 2 months | Multiple organs involvement | *Not mentioned* | *Not mentioned* | Craniocervical junction, epidural | Partial resection, steroids and chemotherapy | >12 months, stable |
| Purav P, *et al* | 18/male | Myelopathy | Multiple organs involvement | *Not mentioned* | *Not mentioned* | Cervical, epidural | Near total excision | 10 months, stable |
| Chen KT, *et al* | 62/male | Buttock pain and cauda equina syndrome | Multiple organs involvement | *Not mentioned* | *Not mentioned* | Sacrum, dura-based | Laminectomy and biopsy | 4 years, no recurrence |
| Yip CC, *et al* | 68/male | Back pain and myelopathy | Multiple organs involvement | No | *Not mentioned* | Thoracic, epidural | Laminectomy and lesion resection | 75 months, progression of lesion on other organ |
| Wu M, *et al* | 35/male | No spine-related symptoms | Multiple organs involvement | *Not mentioned* | Normal | Lumbar, intrabony | Watch and wait | 5 years, stable |
| Andriko JA, *et al* | 51/male | Acute myelopathy | *Not mentioned* | *Not mentioned* | *Not mentioned* | Thoracic, epidural | Resection | 3 months, no recurrence |
| Andriko JA, *et al* | 35/male | Myelopathy for 4-5 weeks | *Not mentioned* | *Not mentioned* | *Not mentioned* | Thoracic, intramedullary | Resection | 12 months, stable |
| Hollowell JP, *et al* | 78/male | Myelopathy for 6 months | *Not mentioned* | No | Elevated ESR | Multiple, epidural | Resection | 18 months, stable |
| Kelly WF, *et al* | 45/female | Myelopathy | Multiple organs involvement | *Not mentioned* | Elevated ESR and CRP | Thoracic, intradural | Lesion resection, steroids, radiotherapy and chemotherapy | 36 months, progression of lesions on other organ |
| Katz DS, *et al* | 12/male | Myelopathy | Multiple organs involvement | No | *Not mentioned* | Upper cervical, intracanal | Lesion resection | 8 years， new lesions on other organs |
| Unni KK, *et al* | 16/female | Sacral pain for 4 months | *Not mentioned* | Yes | Elevated ESR | Sacrum, intrabony | Watch and wait | New lesions on other sites |
| Haas RJ, *et al* | 13/female | Myelopathy | Multiple organs involvement | Yes | Elevated ESR and neutrophilia | Cervical and thoracic, epidural | Steroids and chemotherapy | 4 months, stable |
| Kessler E, *et al* | 53/male | Myelopathy for 2 months | *Not mentioned* | Yes | Elevated ESR | Throacic, epidural | Laminectomy and lesion resection, radiotherapy | 2 months, stable |
